# Communication with face masks during the COVID-19 pandemic for adults with hearing loss

**DOI:** 10.1186/s41235-022-00376-8

**Published:** 2022-03-21

**Authors:** Brenda T. Poon, Lorienne M. Jenstad

**Affiliations:** 1 Wavefront Centre for Communication Accessibility, 2005 Quebec Street, Vancouver, BC V5T 2Z6 Canada; 2 School of Population and Public Health, University of British Columbia, 440 - 2206 East Mall, Vancouver, BC V6T 1Z3 Canada; 3 School of Audiology and Speech Sciences, University of British Columbia, 4th Floor Friedman Building, 2177 Wesbrook Mall, Vancouver, BC V6T 1Z3 Canada

**Keywords:** COVID-19, Face masks, Deaf, Hard of hearing, Hearing loss, Communication, Social interaction, Barriers, Speechreading, Accessibility

## Abstract

Face masks have become common protective measures in community and workplace environments to help reduce the spread of severe acute respiratory syndrome coronavirus 2 (SARS-CoV-2) infection. Face masks can make it difficult to hear and understand speech, particularly for people with hearing loss. An aim of our cross-sectional survey was to investigate the extent that face masks as a health and safety protective measure against SARS-CoV-2 have affected understanding speech in the day-to-day lives of adults with deafness or hearing loss, and identify possible strategies to improve communication accessibility. We analyzed closed- and open-ended survey responses of 656 adults who self-identified as D/deaf or hard of hearing. Over 80% of respondents reported difficulty with understanding others who wore face masks. The proportion of those experiencing difficulty increased with increasing hearing loss severity. Recommended practical supports to facilitate communication and social interaction included more widespread use of clear face masks to aid lip-reading; improved clarity in policy guidance on face masks; and greater public awareness and understanding about ways to more clearly communicate with adults with hearing loss while wearing face masks.

## Significance statement

The rapidly evolving severe acute respiratory syndrome coronavirus 2 (SARS-CoV-2) and emergence of coronavirus disease in 2019 (COVID-19) have been unsettling, changing the daily lives of people around the globe. Around the world, face masks are routinely recommended or required as one of the preventive measures to stop the spread of SARS-CoV-2. Our study findings highlighted that although people with hearing loss or deafness view face masks as essential health and safety measures to protect against SARS-CoV-2 infection, consistent use also makes communication very difficult, frustrating, and stressful on a daily basis, especially for those with greater severity of hearing loss. Particularly problematic are opaque face masks that severely disrupt communication as they block access to key visual cues and auditory information important for hearing and understanding speech. Our study findings revealed that there were marked discrepancies in recommended best practices for safe, clear communication with face masks. Increased and improved practice and policy guidance are needed, particularly if widespread use of opaque face masks continues to be the norm. Clear or transparent face masks may offer one potential solution; however, the success of this measure in facilitating communication and social interaction relies on mask quality improvement, more widespread availability, and broader societal use.

## Introduction

The emergence of severe acute respiratory syndrome coronavirus 2 (SARS-CoV-2) and coronavirus disease 2019 (COVID-19) pandemic have led to a significant increase in public health measures around the world, with face coverings now being routinely recommended or required in many settings as one of the preventive measures to stop the spread of the virus (e.g., Czypionka et al., 2021). Face masks have the potential to disrupt communication through two mechanisms: loss of visual cues and loss of auditory cues. Opaque face masks block access to the important visual cues necessary for speech understanding; mouth movement and other facial cues carry crucial cues for speech recognition for both spoken and signed language (Atcherson et al., 2017; Goldin et al., 2020).

Visual cues provided by lip and mouth movement (e.g., Grant & Bernstein, 2019) contribute to speech recognition; visual speech information improves speech recognition in noisy environments by up to 40% for listeners with normal hearing (Ma et al., 2009; MacLeod & Summerfield, 1987; Ross et al., 2007). These cues are particularly important for listeners with hearing loss (e.g., Erber, 1975; Miller et al., 2017; Woodhouse et al., 2009), as they carry rich information about consonants that can be difficult to hear by listeners with high-frequency hearing loss (Erber, 1975). Facial cues are also important in signed languages, where facial expressions carry information about prosody, intonation, and phrase boundaries (e.g., Sandler, 2012). Appropriately worn face masks cover the nose, mouth, and chin and remove access to those supplemental visual cues.

Face masks also attenuate acoustic speech information, with the amount of attenuation differing across mask and material type. Information-bearing high frequencies (2000–7000 Hz) are attenuated 3–4 dB by conventional surgical masks and up to 12 dB by N95 masks (Goldin et al., 2020). Variations in non-medical (cloth) masks can attenuate high frequencies above 1000 Hz by 4–12 dB, depending on material, weave, and number of layers (Corey et al., 2020). As expected, these acoustic alterations have negative consequences on speech recognition even for listeners with normal hearing (e.g., Bandaru et al., 2020; Yi et al, 2021). Hard of hearing listeners report difficulties understanding those wearing face masks (e.g., Saunders et al., 2021), with consequences for social interaction and participation. Given the combination of loss of visual and acoustic cues due to face coverings, it is expected that a listener with hearing loss would have difficulty participating in conversation with others wearing face masks, and it is likely that this difficulty would increase with increasing degree of hearing loss.

### Purpose

We conducted a large survey to understand the impact of the COVID-19 pandemic and related preventive measures on the daily lives of people in Canada who are D/deaf,^1^ DeafBlind, or hard of hearing, with particular focus on communication accessibility and access to information. From that survey, there was a prominent theme of face masks throughout the open-ended survey responses. Thus, the purpose of this report is to analyze and describe the subset of survey responses related to consequences of face masks for this population. At the time of the survey, most jurisdictions in Canada had a mask mandate in place for all indoor gatherings, with rare exceptions where masks were at least recommended. Although clear face masks were available, they were not in widespread use. All respondents, therefore, had experience with communicating with others wearing opaque face masks.

## Method

We collected data about face masks and their influences on communication as part of a nationwide, cross-sectional survey to better understand the impacts of the pandemic on the daily lives of people who are D/deaf or hard of hearing in Canada. We developed the *Impacts of COVID-19 on Communication Accessibility for Adults with Hearing Loss Survey* (see “Appendix”) using Qualtrics Survey software and administered it online after research ethics approval was obtained from the University of British Columbia’s Behavioural Research Ethics Board (Reference no. H20-03937). Consistent with our mixed methods approach, we designed a survey that included 55 closed-ended and open-ended questions, with additional branching questions that were response dependent, about the extent that: (1) information about COVID-19 is accessible; (2) COVID-19 health and safety protective measures have influenced communication accessibility; and (3) participants have been affected socially, mentally, and financially by COVID-19 and the COVID-19 response. In this paper, we present selected findings about face masks based on our analysis of a subset of the larger survey data set.

Two long-standing organizations in Canada that specialize in providing services and support to adults who are D/deaf or hard of hearing distributed the survey hyperlink to their respective client and member networks. In order to participate, individuals were required to be 19 years of age or older, self-identify as D/deaf or hard of hearing, have access to a computer or mobile device with an internet connection, and be located in Canada. Prior to starting the survey, each individual accessed an online project information page noting that participation was voluntary and that completion of the survey was an indication of consent. The online survey was available from March 30 to April 9, 2021, in written English and in American Sign Language (ASL) through an embedded link to video with ASL translation of each question.

We analyzed the survey data using a combination of an inductive qualitative content analysis approach (Hsieh & Shannon, 2005), accompanied by relevant descriptive statistical analysis. To analyze the qualitative data on face masks, all text stemming from the open-ended survey questions that made reference to “mask” or “masks” was identified, organized into its own data set, then open coded line-by-line using NVivo (Release 1.5; QSR International Pty Ltd., 2021). Data extracts were continually compared and contrasted conceptually, not by demographic background of the participants. Codes were refined accordingly using sub- or child codes, integrated into new codes, or combined with other codes to create overarching categories that subsumed multiple codes. The two authors engaged in peer debriefing periodically throughout the analysis to collectively review the emerging codes with respect to the data extracts. This analytic process continued until no new codes emerged.

The responses to the relevant closed-ended survey questions (i.e., question numbers 6, 23, 52 in “Appendix”) were analyzed using Chi-square tests to examine the underlying distributions across responses for two specific pre-planned analyses using SPSS v 27.0. The primary question of interest was whether there was any relationship between self-reported degree of hearing loss and self-reported ease/difficulty understanding others wearing face masks. Only data for participants reporting hearing loss were used for this analysis. The secondary question was whether there was any relationship between self-reported ease/difficulty understanding others wearing face masks and self-reported change in social interaction. Data for all participants were used for this analysis. It was hypothesized that increased degree of hearing loss would be associated with increased difficulty understanding others, which in turn would be associated with reductions in social interaction. Significant Chi-square results were analyzed with paired comparisons, with *p*-values adjusted using the Bonferroni method for multiple comparisons, with family-wise error set at 0.05.

## Results

We received 656 completed surveys. Age, gender, and degree of self-reported hearing loss of the respondents are shown in Table 1. Of those adults who responded, 60% self-identified as female, 39% male, and 1% as other. The majority of respondents (82%) were between 51 and 90 years old, very fluent in written English (87%), and resided in a large urban population center (69%). A range of socio-economic backgrounds was represented, with 20% reporting a household income of under $30,000 CAD; 30% between $30,000 and $59,999; 24% with $60,000 to $89,999; and 26% with $90,000 and above. With respect to highest level of education, approximately half of respondents held a university degree (51%).

**Table 1:** Number of participants with each degree of self-reported hearing loss by age group and gender

Degree of self-reported hearing loss	18–30 years		31–50 years		51–70 years		71–90 years		90+ years		Total
	M^a^	F^b^	M	F	M	F	M	F	M	F	
Mild	1	2	1	7	8	11	13	15	0	0	58
Moderate	2	7	4	19	32	35	62	61	1	3	226
Severe	4	5	2	15	18	54	39	31	1	3	172
Profound	2	7	6	12	20	56	17	21	0	2	143
Total	9	21	13	53	78	156	131	128	2	8	599

Demographic characteristics of the subset of participants who responded to all of the gender, hearing loss, and age questions, excluding 3 individuals who reported “other gender,” 6 individuals who reported no hearing loss, 2 individuals who responded “prefer not to answer” for age, and 46 additional participants who did not complete one or more of the questions regarding gender, hearing loss, or age. Degree of hearing loss is shown as a function of age group and gender. Cell values show the number of participants with those characteristics

^a^M = Male

^b^F = Female

With respect to hearing and communication, 78% of the sample identified as hard of hearing, 9% as Deaf, 3% as oral deaf, and < 1% as DeafBlind. A further 10% of the sample identified as “none of the above” or “other,” with “other” explained by the respondents in a variety of ways, including “deafened” and “have cochlear implant” and making reference to their degree of loss. Participants were asked about their primary mode of communication, with 90% of respondents indicating “speaking,” 7% indicating “both speaking and sign language” and 3% indicating “sign language.”

### Face masks as barriers to social interaction

**Fig. 1:**
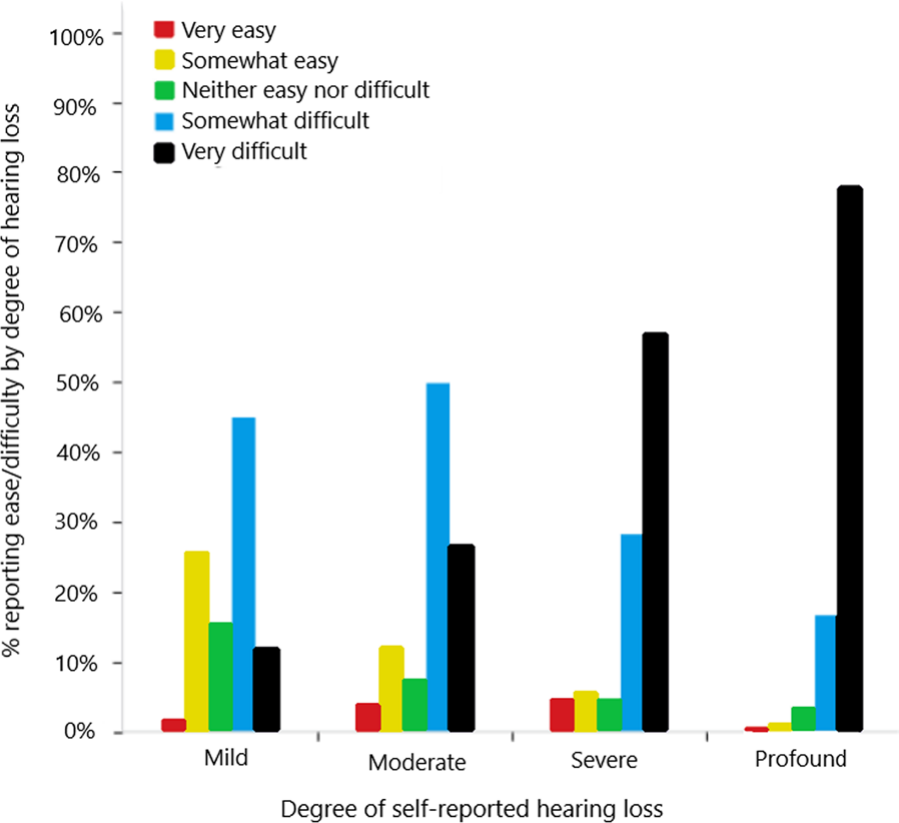
Reported difficulty of understanding those wearing face masks by degree of self-reported hearing loss

In response to the question “How easy or difficult has it been for you to understand others who are wearing face masks?” (see question number 23 in “Appendix”), we found that 81% of respondents reported difficulty with understanding others who wore face masks. The bars in Fig. 1 show the proportion of respondents within each hearing loss category who responded with each category on the ease/difficulty of understanding scale. Chi-square analysis confirms the trend apparent on the figure, that there is a significant relationship between ease of understanding others who are wearing face masks and degree of self-reported hearing loss (*Χ*^2^(12, 641) = 158, *p* < 0.001). The significant finding was further analyzed with pairwise z-tests corrected with the Bonferroni correction for multiple comparisons, keeping family-wise error at *p* < 0.05. Specifically, respondents with mild hearing loss responded “Somewhat easy” and “Neither easy nor difficult” significantly more often than respondents with any other degree of hearing loss. Those with moderate hearing loss responded “Somewhat difficult” significantly more often than respondents with severe or profound hearing loss. Respondents with severe or profound hearing loss responded “Very difficult” more often than those with mild or moderate hearing loss.

According to participants’ open text responses (for question numbers 53, 54, and 55 in “Appendix”), key ways that face masks were barriers for social interactions for participants who are D/deaf or hard of hearing included the following: (1) Face masks hindered speech comprehension; (2) Wearing face masks with hearing aids was problematic; and (3) Communication with masks was stressful. As one participant highlighted,^2^ “The masks is extremely difficult to hear people, never mind trying to find out who is speaking. People assume you can hear them when you can’t. I understand why we all must wear a mask and social distance but it certainly adds to the struggles of people with hearing loss.”

### Face masks hindered speech comprehension

Primary reasons that masks made it difficult to understand others were that they (1) interfered with a D/deaf or hard of hearing person’s ability to lipread and see facial and visual cues; and (2) degraded a speaker’s speech particularly in terms of volume and clarity. First, with respect to lipreading, participants noted their typical reliance on it for understanding others pre-pandemic and that masks added a significant barrier to communication because it directly interfered with their ability to speechread and obtain facial cues (e.g., facial expressions). As one participant stated, “Lipreading is a skill I heavily rely on and masks really impacted my ability to understand the other person…. Sometimes I am almost in tears trying to understand what is being said and feeling lost in a sea of sounds that do not connect. It can feel very isolating.”

Some participants commented that use of hearing aids or cochlear implants in and of themselves was not always sufficient to facilitate hearing or understanding others with masks, because they still were prevented from lipreading/speechreading. For some participants, the situation could become stressful and frustrating, particularly if participants perceived inflexibility from others to accommodate the needs of people who are D/deaf or hard of hearing. For example, a participant commented, “I actively avoid having to communicate with people because it's so frustrating and there is no accommodation in terms of pulling down masks (I speech read) so I shopped at stores that had self-serve check-outs, even if they were farther away from home on foot.”

A second way that masks affected communication was through their negative impacts on speech clarity and volume. Others’ speech sounded not only garbled, muffled, and distorted through the masks, but also quieter for each participant in the interaction, making listening and communication very challenging. Struggling to hear speech clearly required extra effort that could be tiring and frustrating, as reflected by this participant’s comment: “Through the mask their voices sound garbled and it is frustrating. I get a headache from struggling to hear them.”

### Wearing face masks with hearing aids was problematic

Communication with others also became much more challenging for some individuals who are D/deaf or hard of hearing who indicated that “wearing hearing aids and a mask over the ears is a pain” because of the incompatibility of wearing hearing aids at the same time as a face mask. As one participant noted, “I cannot wear my hearing aids out-and-about—the conflict of wearing them over my ears and then the mask getting in the way.”

Because wearing a face mask was mandatory for public health and safety, some participants opted to go without hearing aids and wear just the face mask instead, as indicated by one participant’s comment, “Wearing a mask at work and shopping does not work well with hearing aids, so I've had to forego wearing them for now.” Some participants also reported fear of losing their hearing aids while wearing face masks as another reason for not wearing their hearing aids on outings.

### Communication with face masks was stressful

Some participants reported the following feelings about use of face masks by communication partners: Stress, frustration, and feeling excluded and isolated. As one participant stated, “While I do text chat a lot, I miss seeing people and hearing their voices (with my limited hearing). I have met up with friends in public spaces a few time but they have worn masks, which completely excluded me and made me feel terrible.”

To further contribute to the difficulties, there was also a perceived lack of understanding from others about ways that masks create barriers to communication. Moreover, there was a call for others to be more patient and aware of the communication challenges associated with wearing a mask, particularly for those with hearing loss, and also to learn ways to facilitate communication. This included asking others to “Consider how difficult it is to hear and understand someone while wearing a mask” and to “Be aware that masks are a barrier.” Businesses could play a key role in promoting greater awareness and best practices for communication by providing their staff with sensitivity training and more specific strategies to support their interactions with customers who are D/deaf or hard of hearing.

For some, the stressful nature of social interactions resulted in avoidance of situations that involved communication with others wearing face masks and general reluctance to go to public spaces. One participant commented, “Those masks are so hard to hear through! I avoided leaving the house at all costs because interacting with the public was so much harder and it was hard to hear/understand.” This was echoed by others, including one who stated, “I can’t hear through masks so I avoid social interactions,” and another who wrote, “I have become very anxious about my interactions outside of the house, particularly in places where people are wearing masks or where there are plexiglass barriers. I usually ask my husband to go into those places for me.”

**Fig. 2:**
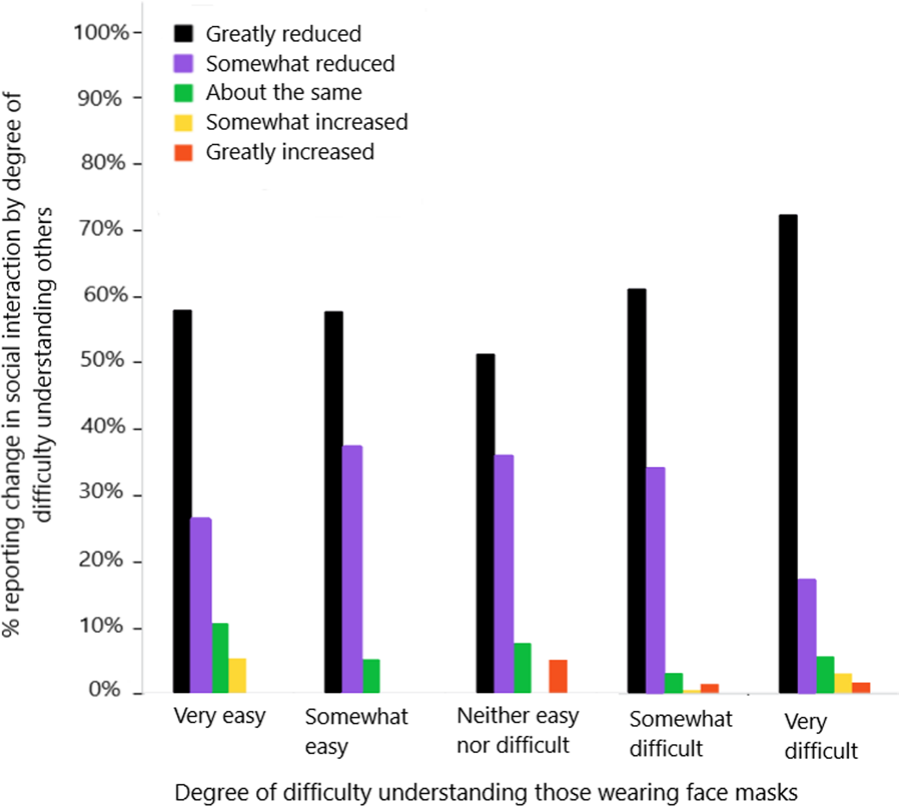
Reported change in social interaction as a function of difficulty understanding others wearing face masks

Figure 2 illustrates responses to the question “How have your social interactions or connections changed as a result of COVID-19 and the health restrictions?,” where the possible responses on a 5-point scale ranged from “Greatly reduced” to “Greatly increased” (see question number 52 in “Appendix”). The responses are plotted as a function of difficulty understanding those wearing face masks. The percentages on the graph are the percentage of respondents within each category of face mask difficulty who chose each category of change in social interaction. As expected due to pandemic restrictions, regardless of difficulty understanding, most participants responded that social interactions were reduced. However, there is a statistically significant relationship between level of social interaction and ease of understanding others wearing face masks (*Χ*^2^(16, 646) = 39.1, *p* < 0.001). The significant finding was further analyzed with pairwise z-tests corrected with the Bonferroni correction for multiple comparisons, keeping family-wise error at *p* < 0.05. Specifically, those who reported that it was *very difficult* to understand those wearing face masks were significantly more likely (*p* < 0.05) to report greatly reduced social interactions than those who had less difficulty with face masks.

### Strategies for communication with face masks

Participants put forward several strategies that others wearing face masks could use to improve communication with people with hearing loss or deafness during the pandemic. Participants were first asked “Think of a situation where you experienced any difficulty understanding a person wearing a mask. Which of the following actions by the other person would have made it easier for you to hear or understand them? Rank the strategies in order, from most preferred to least preferred.” (see question number 25 in “Appendix”). The possible actions are shown in Table 2, with the cells displaying the percentage of respondents who ranked the action as first, second, third, etc. most-preferred action. The actions are listed in descending order, with the overall most preferred action listed first (“Stepped back and lowered their mask”). The overall ordering was determined by a weighted average of the responses that incorporated respondents’ first, second, and third overall preferred actions, with the most preferred action given the highest weighting, according to the formula:

$$\left( {\% P1 \times 3 + \% P2 \times 2 + \% P3} \right) \div 3$$

where %*P*1 is the percentage of respondents who ranked that strategy as most preferred, and %*P*3 is the percentage of respondents who ranked that strategy as third overall preferred.

**Table 2:** Rank ordered communication strategies that those wearing masks could do to make understanding them easier

Action	Weighted preference for top 3 choices (%)	Rank order of responses							
		1 (%)	2 (%)	3 (%)	4 (%)	5 (%)	6 (%)	7 (%)	8 (%)
Stepped back and lowered their mask	48.8	35.0	15.8	10.0	15.1	8.1	3.8	3.5	8.9
Spoke more clearly	45.7	17.7	30.9	22.2	12.6	9.9	4.8	1.2	0.8
Spoke louder	36.7	20.0	15.1	19.9	11.3	9.2	9.5	8.7	6.2
Used a clear mask	24.7	12.2	13.6	10.5	12.5	24.3	14.3	8.2	4.4
Spoke slower	18.0	4.1	10.2	21.4	25.6	14.8	12.0	7.9	4.1
Wrote down information	13.8	7.1	5.9	8.5	11.2	16.9	34.5	12.0	3.9
Used a speech to text app	7.3	1.8	5.4	5.6	6.1	8.1	12.8	43.5	16.8
Used a device that amplified their voice	4.9	2.1	3.1	2.0	5.6	8.9	8.4	15.1	54.8

Each cell shows the percentage of respondents who chose each response at each rank order, where 1 was most preferred and 8 was least preferred. The options are listed in order of overall preference, using a weighted average (see text for more details)

Participants were then given the opportunity to elaborate or offer additional strategies. In their open text responses, participants highlighted the importance of using a combination and range of preferred strategies rather than any single strategy. For example, one participant recommended that others communicating with people who are D/deaf or hard of hearing should “1. Face people when you speak to them! 2. If you know you are going to speak with a HOH^3^ person, use a clear face mask if you can, or at very least, make sure you are enunciating well and speaking at a slightly increased volume.”

Some felt that clear face masks should be more widely used and mandated, as indicated by this participant, “We need to see your whole face while communicating with us! Mandate masks that allow mouths to be seen would be extremely helpful.” Similarly another noted, “I read lips, this was a habit that I formed before I got aides and it continues. So masks are a hindrance to me. I would like to see people wear clear plastic visors so I can see their mouths.”

Though recommendations for wider availability and public use of clear face masks were evident in participants’ comments, some indicated that their experiences with clear face masks were not always positive, with a need for improvements in effectiveness. A participant noted, “I wish there were good clear masks that didn't fog up and they were more normalized so more people would wear them,” with another also indicating, “The clear masks are no better because they fog up or you cannot see all of their mouth to be able to speech read.”

Another recommended strategy to enable lipreading and improved access to facial cues was for others to step back at a safe distance then lower or remove their face masks to facilitate communication, as indicated by this participant, “I read lips. If possible, step back and remove mask so I can read/hear what you are saying.” Others similarly recommended, “Mask free at a safe distance” or “Please consider stepping back and removing masks to interact.”

Some participants expressed frustration when requests for others to lower their masks were refused. One participant indicated, “As with shopping and other services, the plexiglass and masks that are worn by service providers makes it very difficult to understand. When I noted that I was hard of hearing and if they could pull down their mask I was almost always refused and told it was provincial guidelines. It creates embarrassment to hold up lines and have someone repeat-scream because you cannot hear.” In these cases, lowering the masks while stepping back and social distancing was viewed by these participants as sufficient and acceptable practice to prevent exposures to SARS-CoV-2, as noted by this participant, “there is no risk to you if you lower your mask at a safe distance to convey information,” and also another, “Remember, physical distancing is very effective in preventing contagious viruses like Covid. Please consider stepping back and removing masks to interact.” Some felt that it would be beneficial to introduce a policy in public establishments that enabled flexibility for staff and service providers to step back and lower their masks to foster communication. A participant recommended for decision-makers to “make accommodations clear so that service providers and businesses are aware if they can pull down their masks without repercussions.”

Support for the strategy of temporarily lifting or removing face masks was not universal, however, as there was a clear tension among participants about the need to choose between protecting health and safety by wearing face masks at all times or possibly increasing risks of health and safety to improve communication and understanding. This tension was evident in this participant’s comment, “Look at me, face me clearly, use gestures if you need to, speak loudly and clearly. Yes pulling your mask down helps me hear you much better, but I don't love it from a public health perspective at all” and echoed by another, “It all feels very hard. I feel like I have to choose between staying safe with everyone wearing masks OR no masks and distance but it doesn't feel safe. So I choose to stay home until I can confidently, responsibly understand others.”

There was recognition among participants that not all people would feel comfortable, safe, or able to step back and lower their masks to foster communication. If stepping back and lowering the face mask did not feel safe by others or was not permissible (i.e., in spaces where masks were mandatory), then participants put forward alternatives, as evident in this comment, “I read lips. If possible, step back and remove mask so I can read/hear what you are saying. If not, possible please speak clearly and slowly. Otherwise, have it written out. Above all, please be patient with us. We are ALL frustrated.” A similar recommendation made by another was for others to “Be patient and if you don’t feel or is not safe to step back and lower your mask to communicate, be flexible and accepting if asked to write down what they are trying to convey.”

Some participants expressed in no uncertain terms that lowering or removing the mask was not a recommended strategy. For these participants, maintaining and protecting personal and public health and safety was essential. As one participant commented, “DO NOT under any circumstance feel you have to take down your mask, step out from behind plexiglass, or do anything else that would put you at risk. I appreciate you trying, by speaking more loudly, slowly, distinctly (and almost everyone does), but if it's a choice between me hearing one interaction or anyone being put at heightened risk, the first thing to remember is that we're in a global health emergency. If the conversation really matters, we'll figure out together how to make it work.”

As much as face masks introduced significant barriers to communication and day-to-day social interactions, participants also viewed them as a necessary measure to protect against infection. Participants reported feeling unsafe with fears of infection when in public spaces where individuals were not wearing face masks, such as when shopping or taking the bus. Public transit was highlighted as a high risk context where participants experienced fears of infection and called for greater cooperation or compliance amongst the general public to wear face masks and maintain social distance, as well as for service/business operators to be much more consistent and strict in their enforcement of these measures. As one participant commented, “I am nervous about being in a setting where there are a lot of people and some choose not to wear masks. There is no enforcement of mask wearing on the seabus and on buses, so I don't feel safe.” Proper mask wearing of others on the bus was also problematic, where one noted a “fear of those who don't practice social distancing and don't wear masks properly or at all,” with similar comments from another noting that they “… do not feel safe on transit yet—see too much crowding and people with masks under their chins.” For some, exposures to these potentially unsafe environments lead to inconveniences and disruptions to daily living in order to minimize risks, such as exiting the bus prior to one’s destination or avoidance of public transit altogether, such as with this participant, “I stopped almost entirely taking transit; I don’t know if there’s a way to ensure that all people wear masks.”

## Discussion

Our findings are consistent with a growing body of previous research indicating that face masks created communication difficulties in the day-to-day lives of adults who are D/deaf or hard of hearing during the COVID-19 pandemic (Homans & Vroegop, 2021; Saunders et al., 2021). Experiences of difficulty understanding others with face masks became more pronounced with increasing severity of hearing loss. Degraded speech understanding and limited access to visual cues from the lips and face made communication particularly challenging (Homans & Vroegop, 2021; Naylor et al., 2020; Saunders et al., 2021) and resulted in various emotional and behavioral consequences, such as negative emotional reactions (e.g., stress, frustration) and also in some cases avoidance of situations or environments that involved communication (Saunders et al., 2021). Day-to-day wearability of face masks also proved challenging for some, especially those with hearing aids. Wearing face masks simultaneously with hearing aids was not always compatible or comfortable, which is consistent with reports from other studies (Naylor et al., 2020; Trecca et al., 2020).

Notwithstanding these difficulties participants experienced with face masks, there was still recognition of the role of face masks in protection against SARS-CoV-2 infection. Similar to findings from a previous survey on impacts of face masks on communication during COVID-19 (Saunders et al., 2021), participants in the present study indicated the importance of face masks as a public health measure; yet, some participants were ambivalent about their widespread societal use. In the present study, there were mixed opinions about the strategy of requesting others to temporarily lower or remove face masks to foster communication. Some felt this flexibility in approach was appropriate and safe practice, particularly if the communication partner stood at a safe distance, whereas others felt that keeping face masks on was necessary for minimizing risk of infection, even if it meant the cost of poorer communication with others.

At the time the survey was conducted, mask mandates were in place for all indoor spaces across almost all jurisdictions in Canada, except Northwest Territories and some regions of Nunavut. The public health orders regarding the mask mandate generally allowed for exemptions to wearing the mask; e.g., for children under the age of two years, or people who could not don or remove their own masks. Many jurisdictions allowed exemptions as needed to accommodate individuals with disabilities (e.g., Ontario, Nova Scotia) but did not explicitly mention hearing loss nor provide clarity about who could remove the mask. Only two of thirteen provinces and territories explicitly allowed an exemption to the mask policy for communicating with someone who is D/deaf or hard of hearing: British Columbia and Saskatchewan, although the latter was restricted to apply only during the provision of personal support services. Thus, experience with the strategy of temporarily removing the mask in public indoor spaces may have been limited at the time of the survey. Given the divergent participants’ views of best practices for communication while wearing face masks, there is clearly a need for increased practice and policy guidance about effective strategies for face mask use that integrate BOTH high-level protection for public health and safety AND clear, effective communication (Giovanelli et al., 2021).

Strategies to enhance communication with face masks that participants put forward in the present study were consistent with recommendations from others (Chodosh et al., 2020; Deardorff et al., 2021; Schlögl & Jones, 2020), such as promoting greater public awareness of masks as barriers to communication and use of basic strategies to aid communication, such as speaking slower, slightly increasing speech volume, and incorporating written or typed text. Use of clear or transparent face masks offer potential for improved communication, as they support listeners’ access to speakers’ facial cues for communication, while still providing some level of protection from SARS-CoV-2 infection (Atcherson et al., 2021; Thibodeau et al., 2021). Many survey participants called for widespread use of clear face masks, based on expected benefits for facilitating communication. It is important to note, however, that there were few reports of first-hand experiences with clear face masks, as these masks are not yet widely manufactured or used (Chodosh et al., 2020; Sheik-Ali et al., 2021). In fact, the few experiences that were described indicated problems of masks fogging or a “fog effect” owing to moisture created when speaking (Thibodeau et al., 2021), which in turn cancelled out the expected benefit of improved access to facial cues. Relative to non-transparent masks, clear face masks have been found to perform poorly acoustically, thus contributing to communication difficulties (Atcherson et al., 2021; Corey et al., 2020). Future community-based investigation about the day-to-day experiences of both speakers and listeners using clear face masks to communicate in diverse real-world contexts would be a useful complement to previous laboratory-based experiments. Also worthwhile would be further exploration of potential innovations or supplemental strategies (e.g., pairing clear masks with amplification technologies; see Corey et al., 2020) to improve the effectiveness of clear face masks, both in terms of acoustic performance and consistent access to facial cues.

With respect to study limitations, our survey was administered online, meaning that access was easiest for those segments of the target population who had internet connectivity and were also comfortable navigating an online survey. Our method of sampling did not enable purposeful selection of a cross-section of participants from diverse backgrounds, though our sample consisted of participants with a range of socio-economic backgrounds, as well as reasonably varied composition in terms of gender and age. We asked respondents to estimate their self-rated hearing loss using the standard clinical descriptors (i.e., mild, moderate, etc.) and it is possible that they would not know which descriptor to use, resulting in uncertainty regarding those responses. However, the groups to whom the survey was sent would be mainly comprised of people likely to have some understanding of the clinical terms through routine hearing assessments and multiple educational opportunities. The distribution of hearing status among survey participants reflected strong representation from adults who self-identified as “hard-of-hearing;” however, there were comparatively fewer surveys received from those who self-identified as D/deaf or DeafBlind. The questions were provided in both English and ASL, but the only response options for the open-ended questions were in English, which may have been seen as a barrier to participation.

## Conclusions

As expected, face masks interfere with communication, social connection, and even tasks of daily living (e.g., buying groceries). Findings from our study indicate that the vast majority of people who are D/deaf or hard of hearing reported difficulties understanding others with face masks during the COVID-19 pandemic; and, notably, those with more severe self-reported hearing loss were also more likely to report greater difficulties in communication. Additional applied community-based research is needed to better understand users’ day-to-day experiences and perspectives, both of speakers and listeners, about the effectiveness of known best practices for addressing communication difficulties with face masks in diverse real-world contexts. Future study could also involve purposeful sampling and age- and gender-based analysis and also delve further into identifying and evaluating the range of potential solutions involving clear face masks and wearing face masks simultaneously with hearing aids. Further research would also be beneficial on strategies for promoting widespread public awareness about improving clarity of communication with face masks while still protecting public health and safety. A unique contribution of our study is that it highlighted ways that the D/deaf or hard of hearing individuals’ preferred strategies for coping with the communication challenges with face masks during the pandemic (e.g., asking others to lower or remove face masks) were intertwined with ways they balanced and navigated the tension between maintaining safety and fostering clear communication. Our findings are suggestive of the need for clearer policy and guidance that balance both effective communication *and* safety from a public health perspective. Clarity is needed from decision-makers about the extent that policy guidance on face masks is flexible within certain contexts or when other protective public health measures are applied (e.g., communication at a safe distance or behind physical partitions).

## Data Availability

The datasets used and/or analyzed during the current study are available from the corresponding author on reasonable request.

## Appendix: Survey: Communication accessibility for adults with hearing loss during the COVID-19 pandemic

The rapidly evolving coronavirus (COVID-19) situation has been unsettling, changing daily lives of people around the globe. There is a great deal of uncertainty and we are all relying on news briefs, social media, health videos and information provided electronically to ensure we have received the most current health updates. This information is being updated on an hourly basis and it is important the content reaches all Canadians.

The purpose of this survey is to collect information on the impact the pandemic has had on the daily lives of people who experience hearing loss and deafness. This information will help us better understand how the pandemic has been experienced and also how information about the virus and disease can best be delivered to the public.

1. Do you identify as:
  - Male
  - Female
  - Other (please specify)
  - I prefer not to answer
  - Other (please specify):
2. What age category are you in?
  - 18–30 years
  - 31–50 years
  - 51–70 years
  - 71–90 years
  - 90+ years
  - I prefer not to answer
3. What is your household income?
  - Under $30,000
  - $30,000–$59,999
  - $60,000–$89,999
  - $90,000–$119,999
  - Over $120,000
4. What is the estimated population of the area you live in?
  - Small population center (1000–29,999 people)
  - Medium population center (30,000–99,999 people)
  - Large urban population center (100,000+ people)
  - Rural area (all territory outside population center)
5. What is the highest level of education you have received?
  - Less than high school
  - Completed high school
  - Completed a university certificate or diploma below bachelor level
  - Completed bachelor’s degree
  - Completed master's degree
  - Completed doctoral degree
  - Completed registered apprenticeship or other trades certificate or diploma
  - Completed other non-university certificate or diploma (please specify):
6. What is the estimated severity of your hearing loss?
  - No hearing loss
  - Mild
  - Moderate
  - Severe
  - Profound
7. Do you have hearing loss in both ears?
  - Yes
  - No
8. Do you identify as:
  - Hard of Hearing
  - Deaf
  - Oral deaf
  - Deaf Blind
  - None of the above
  - Other:
9. When was your initial onset of hearing loss?
  - At birth
  - Childhood (1–18 yrs)
  - Adulthood (19–64 yrs)
  - Senior (> 65 yrs)
10. What is your primary mode of communication?
  - Speaking
  - Sign language
  - Both speaking and sign language
11. How fluent are you in written English?
  - Very fluent
  - Moderately fluent
  - Somewhat fluent
  - Not at all fluent
12. Do you use hearing aids?
  - Yes—Both ears
  - Yes—Left ear only
  - Yes—Right ear only
  - No

(13) Do you use a cochlear implant?
  - Yes—Both ears
  - Yes—Left ear, hearing aid on right
  - Yes—Right ear, hearing aid on left
  - Yes—Left ear, no hearing aid on right
  - Yes—Right ear, no hearing aid on left
  - No

(14) What is your primary reason for using hearing aids?
  - To hear and understand speech
  - For environmental and speech awareness only
(15) Do you use any of the following assistive devices or systems? Check all that apply:
  - Telecoil
  - Amplified phone
  - Alerting device, e.g., for fire, security, doorbell, alarm, etc.
  - TV assistive listening device
  - Streaming device for phone, tablet, laptop, etc.
  - Remote microphone
  - TTY
  - VCO phone
  - Don’t know
(16) Which of the following have you used to access COVID-19-related information? Check all that apply:
  - News briefings in video format (e.g., television, laptop, tablet)
  - News briefings on radio
  - Media releases in written English language
  - Online COVID-19 self-assessment tool
  - COVID-19 management-related apps
  - Social media (e.g., Facebook, Twitter)
  - Doctor
  - Employer
  - Word of mouth (e.g., friends, family, colleague)
  - Newspaper
  - None: I don’t access COVID-19-related information
  - Other: please specify

[**Branching questions from #16:]

[If participant answers ‘a’ in #16, then jump to this question:]

(i) How easy or difficult has it been for you to understand COVID-19-related information through news briefings in video format (e.g., television, laptop, tablet)?
  - Very easy
  - Somewhat easy
  - Neither easy nor difficult
  - Somewhat difficult
  - Very difficult

[If participant answers ‘b’ in #16, then jump to this question:]

(ii) How easy or difficult has it been for you to understand COVID-19-related information through news briefings on radio?
  - Very easy
  - Somewhat easy
  - Neither easy nor difficult
  - Somewhat difficult
  - Very difficult

[If participant answers ‘c’ in #16, then jump to this question:]

(iii) How easy or difficult has it been for you to understand COVID-19-related information through media releases in written English language?
  - Very easy
  - Somewhat easy
  - Neither easy nor difficult
  - Somewhat difficult
  - Very difficult

[If participant answers ‘d’ in #16, then jump to this question:]

(iv) How easy or difficult has it been for you to understand COVID-19-related information on the online COVID self-assessment tool?
  - Very easy
  - Somewhat easy
  - Neither easy nor difficult
  - Somewhat difficult
  - Very difficult

[If participant answers ‘e’ in #16, then jump to this question:]

(xxii) How easy or difficult has it been for you to understand COVID-19-related information through news COVID management-related apps?
  - Very easy
  - Somewhat easy
  - Neither easy nor difficult
  - Somewhat difficult
  - Very difficult
(xxiii) Which of the following communication accessibility supports do you use? Check all that apply:
  - Captioning
  - Sign Language Interpreting
  - Visual Relay Service
  - Speech to Text apps or programs
  - Described video
  - Text enhancements for the blind
  - DeafBlind Intervenor
  - None of the above
(xxiv) Which of the following COVID-19 management resources have you accessed? Check all that apply:
  - Published guidelines by provincial health ministry
  - Published guidelines by federal health ministry
  - Published guidelines by Centre for Disease Control (CDC)
  - None: I don’t access COVID-19 management resources.
  - Other: please specify:
  - Not sure

[**Branching questions from #18:]

[If participant answers ‘a’—Provincial Health Ministry guidelines, then jump to these questions:]

(i) How easy or difficult has it been accessing published guidelines by the provincial health ministry about COVID-19 management resources?
  - Very easy
  - Somewhat easy
  - Neither easy nor difficult
  - Somewhat difficult
  - Very difficult
(ii) What could be done to improve access to this information?

(iii) How confident do you feel about the accuracy of the information?
  - Very confident
  - Somewhat confident
  - Fairly confident
  - Not at all confident

[If participant answers ‘b’—Federal health ministry, then jump to these questions:]

(i) How easy or difficult has it been accessing published guidelines by the federal health ministry about COVID management resources?
  - Very easy
  - Somewhat easy
  - Neither easy nor difficult
  - Somewhat difficult
  - Very difficult
(ii) What could be done to improve access to this information?

(iii) How confident do you feel about the accuracy of the information?
  - Very confident
  - Somewhat confident
  - Fairly confident
  - Not at all confident

[If participant answers ‘c’—Centre for Disease Control, then jump to these questions:]

(i) How easy or difficult has it been accessing published guidelines by the Centre for Disease Control (CDC) about COVID management resources?
  - Very easy
  - Somewhat easy
  - Neither easy nor difficult
  - Somewhat difficult
  - Very difficult
(ii) What could be done to improve access to this information?

(iii) How confident do you feel about the accuracy of the information?
  - Very confident
  - Somewhat confident
  - Fairly confident
  - Not at all confident
(19) Have you applied for any COVID-19-related benefits or disability supports?
  - No
  - Yes

[**Branching questions from #19:]

For “Yes” responses in #19 above, branch to Question #20 and #21

For “No” responses, jump to Question #22

(20) Have you experienced difficulty applying for these benefits through on-line portals?
  - No
  - Yes. Please explain reason for difficulty:__________________________________
(21) If you sought assistance to help you apply for these benefits, how was this assistance provided? Check all that apply.
  - Online chat
  - In person appointment
  - Phone call
  - Video call
  - Other: please specify
  - Not applicable: I did not seek assistance to apply for these benefits.

[**Branching questions from Question #21]

[If answer (a) above, then jump to this question:]

How easy or difficult was it for you to apply for these benefits through online chat?

- Very easy
- Somewhat easy
- Neither easy nor difficult
- Somewhat difficult
- Very difficult

[If answer (b) above, then jump to this question:]

How easy or difficult was it for you to apply for these benefits through in person appointment?

- Very easy
- Somewhat easy
- Neither easy nor difficult
- Somewhat difficult
- Very difficult

[If answer (c) above, then jump to this question:]

How easy or difficult was it for you to apply for these benefits through a phone call?

- Very easy
- Somewhat easy
- Neither easy nor difficult
- Somewhat difficult
- Very difficult

[If answer (d) above, then jump to this question:]

How easy or difficult was it for you to apply for these benefits through video call?

- Very easy
- Somewhat easy
- Neither easy nor difficult
- Somewhat difficult
- Very difficult
- What is one change that the government and broadcasters can make in their messaging to improve your ability to access information during critical times like the pandemic?
- How easy or difficult has it been for you to understand others who are wearing face masks?
  - Very easy
  - Somewhat easy
  - Neither easy nor difficult
  - Somewhat difficult
  - Very difficult
- How easy or difficult has it been for you to understand others who are behind plexiglass barriers?
  - Very easy
  - Somewhat easy
  - Neither easy nor difficult
  - Somewhat difficult
  - Very difficult
- Think of a situation where you experienced any difficulty understanding a person wearing a mask. Which of the following actions by the other person would have made it easier for you to hear or understand them? Rank the strategies in order, from most preferred to least preferred. You can drag the options and place them in your preferred order.
  - Stepped back and lowered their mask
  - Spoke more clearly
  - Spoke louder
  - Spoke slower
  - Used a clear mask
  - Wrote down information
  - Used a speech to text app
  - Used a device that amplified their voice
- Think of a situation where you experienced any difficulty understanding a person behind a plexiglass barrier. Which of the following actions by the other person would have made it easier for you to hear or understand them? Rank the strategies in order, from most preferred to least preferred. You can drag the options and place them in your preferred order.
  - Stepped out from behind the barrier and spoke from a safe distance
  - Spoke more clearly
  - Spoke louder
  - Spoke slower
  - Wrote down information
  - Used a speech to text app
  - Used gestures
  - Used a device that amplified their voice
- In the past month, how many video calls (e.g., Facetime, Skype, Zoom) have you made with friends or family?
  - None
  - 1–2
  - 3–5
  -  > 5
  -  > 10
  -  > 20

[**Branching questions from #28]

[If answered b, c, d, e, or f, go to question #29]

[If answered “a,” jump to “Question #27” below]

(29) To what extent did you understand others during video calls?
  - Very high understanding (understood 100% of video call)
  - High understanding (understood 75%)
  - Moderate understanding (understood 50%)
  - Low understanding (understood 25%)
  - No understanding (understood 0%)
(30) What could be improved when communicating with others through video calls? Check all that apply.
  - Internet connection quality
  - Video quality
  - Microphone quality
  - Captioning available
  - Captioning quality
  - Sign language interpreting available
  - Access to recordings
  - Speed of people talking
  - None of the above. No improvements are needed.
  - Other: please specify

(27) In the past month, how many virtual meetings, workshops or webinars have you attended?
  - None
  - 1–2
  - 3–5
  -  > 5

[**Branching questions from above.]

[If answered b, c, or d, go to question #31.]

[If answered “a,” jump to #34 below.]

(31) To what extent did you understand others at these virtual events (meetings, workshops, or webinars)?
  - Very high understanding (understood 100% of virtual event)
  - High understanding (understood 75%)
  - Moderate understanding (understood 50%)
  - Low understanding (understood 25%)
  - No understanding (understood 0%)
(32) What could be improved to make it easier to understand other people at these virtual events? Check all that apply.
  - Internet connection quality
  - Video quality
  - Microphone quality
  - Captioning available
  - Captioning quality
  - Sign language interpreting available
  - Access to recordings
  - Speed of people talking
  - None of the above. No improvements are needed.
  - Other: please specify
(33) Remove
(34) Did COVID-19 restrictions prevent you from obtaining hearing health care services from a hearing specialist such as an audiologist or hearing instrument practitioner?
  - Yes
  - No
  - Not applicable
(35) Did you obtain any hearing or hearing aid services remotely?
  - Yes
  - No
  - Not applicable

[*Branching question from #35]

[If ‘Yes’ above, then move onto next questions, starting at #36]

[If ‘No’ above, then jump to question #38]

(36) How easy or difficult was it for you to access hearing or hearing aid services remotely (i.e., virtually through a video call or phone call)?
  - Very easy
  - Somewhat easy
  - Neither easy nor difficult
  - Somewhat difficult
  - Very difficult
(37) What could be improved in remote hearing or hearing aid services? Check all that apply.
  - Internet connection quality
  - Video quality
  - Microphone quality
  - Captioning available
  - Captioning quality
  - Sign language interpreting available
  - Access to recordings
  - Speed of people talking
  - None of the above. No improvements are needed
  - Other: please specify

What is your likelihood of using remote hearing or hearing aid services again once the pandemic is over?

- Very likely
- Somewhat likely
- Not likely
- Did COVID-19 restrictions prevent you from accessing medical care in your area, such as doctor or nurse in a medical office, health clinic or hospital?
  - Yes
  - No
  - Not applicable
- Did you obtain any medical care remotely (e.g., virtually via a video call or phone call)?
  - Yes
  - No
  - Not applicable

[*Branching question from #39]

[If ‘Yes’ above, then move onto next questions, starting at #40]

[If ‘No’ above, then jump to question #42]

(40) How easy or difficult was it for you to access medical care remotely (i.e., virtually through a video call or phone call)?
  - Very easy
  - Somewhat easy
  - Neither easy nor difficult
  - Somewhat difficult
  - Very difficult
(41) What could be improved in remote general medical services? Check all that apply.
  - Internet connection quality
  - Video quality
  - Microphone quality
  - Captioning available
  - Captioning quality
  - Sign language interpreting available
  - Access to recordings
  - Speed of people talking
  - None of the above. No improvements are needed
  - Other: please specify

What is your likelihood of using remote general medical services again once the pandemic is over?

- Very likely
- Somewhat likely
- Not likely
- As a result of the pandemic, to what extent has your mental health been negatively affected?
  - Not affected
  - Somewhat affected
  - Greatly affected
- Did you obtain any mental health services remotely (i.e., virtually through a video call or phone call)?
  - Yes
  - No
  - Not applicable

[*Branching question from #43]

[If ‘Yes’ above, then move onto next questions, starting at #44]

[If ‘No’ above, then jump to question #47]

(44) How easy or difficult was it for you to access mental health services remotely (i.e., virtually through a video call or phone call)?
  - Very easy
  - Somewhat easy
  - Neither easy nor difficult
  - Somewhat difficult
  - Very difficult
(45) What could be improved in remote mental health services? Check all that apply.
  - Internet connection quality
  - Video quality
  - Microphone quality
  - Captioning available
  - Captioning quality
  - Sign language interpreting available
  - Access to recordings
  - Speed of people talking
  - None of the above. No improvements are needed
  - Other: please specify
(46) What is your likelihood of using remote mental health services again once the pandemic is over?
  - Very likely
  - Somewhat likely
  - Not likely
(47) As a result of the pandemic, did you experience any of the following. Please check all that apply:
  - Job Loss
  - Reduced employment hours
  - Reduced educational opportunities
  - Withdrawal from school/classes
  - Difficulty travelling on public transit
  - Difficulty obtaining groceries or food
  - None of the above
  - Other: please specify

[**Branching questions]

[If answer (a or b) above, then participant moves to this question:]

(48) Tell us more about:
  - why you lost your job or had reduced employment hours.____
  - what support or services would have helped you at that time. ______

[If answer (c or d) above, then participant moves to this question].

(49) Tell us more about
  - Why your educational opportunities were reduced or why you withdrew from school or classes. ______
  - What support or services would have helped you at that time.

[If answer (e) above, then participant moves to this question].

(50) Tell us more about:
  - Why you had difficulty travelling on public transit.
  - What would have helped you to travel on public transit.

[If answer (f) above, then participant moves to this question].

(51) Tell us more about:
  - Why you had difficulty obtaining food or groceries.
  - What would have helped you obtain food or groceries.
(52) How have your social interactions or connections changed as a result of COVID-19 and the health restrictions?
  - Greatly reduced
  - Somewhat reduced
  - About the same
  - Somewhat increased
  - Greatly increased

**Branching questions from #52.

If answered “a” or “b,” go to question #53.

If answered “c,” “d,” or “e” jump to question #54.

(53) If your social interactions and connections have decreased as a result of COVID-19 and the health restrictions, tell us about
  - The barriers to interacting: ________________________________________
  - what it would take to overcome those barriers: ____________________________________
(54) If your social interactions and connections have stayed the same or increased, tell us about
  - the strategies you have used to stay connected with your social network: ___________________________________________________________________________
  - any potential barriers you have had to overcome:___________________________________________________________________
(55) What is one key message that you’d like to share to the public about communicating with people with hearing loss or deafness in a pandemic?

## Abbreviations

D/deaf: Deaf or deaf
HOH: Hard of hearing

## Footnotes

1 D/deaf = Deaf or deaf. D/deaf is used here in reference to those people who self-identify as capital “D” “Deaf” with respect to identifying with Deaf Culture and use of American Sign Language (ASL), or people who self-identify as small “d” “deaf” and may not identify with Deaf Culture and use of ASL.

2 Qualitative data reflect participants’ typed, verbatim responses to open-ended survey questions. To preserve authenticity, we present the data extracts in their original, unedited form.

3 HOH = hard of hearing.
